# The efficacy and safety of remimazolam in painless colonoscopy: a prospective, randomized clinical trial

**DOI:** 10.3389/fmed.2024.1434767

**Published:** 2024-11-20

**Authors:** Haobing Shi, Jinyuan Zhang, Zhiqiang Hu, Qianhao Hou, Qianhua Hu, Zhiguang Dai, Wenjuan Zhou, Dingwu Qi, Yuling Li, Qing Wang, Xiangrui Wang, Lijun Liao, Shuwen Qian

**Affiliations:** ^1^Department of Pain Management, Shanghai East Hospital, School of Medicine, Tongji University, Shanghai, China; ^2^Department of Anesthesiology, Shanghai Children’s Medical Center, School of Medicine, Shanghai Jiao Tong University, Shanghai, China; ^3^Department of Anesthesiology, Ji’an Hospital, Shanghai East Hospital, Medical School, Tongji University, Shanghai, China; ^4^Department of Anesthesiology and Pain Management, Yangpu Hospital, School of Medicine, Tongji University, Shanghai, China

**Keywords:** colonoscopy, remimazolam, propofol, anesthesia, sedation

## Abstract

**Purpose:**

Remimazolam is a new type of ultra-short-effect intravenous anesthetic, that may provide adequate sedation for endoscopy while causing less cardiovascular or respiratory disturbance than propofol. The aim of this clinical study was to compare the efficacy and safety of two different doses of remimazolam with propofol for sedation during colonoscopy.

**Patients and methods:**

225 subjects, aged 18 to 80 years, with American Society of Anesthesiology physical status I-III, were scheduled to undergo colonoscopy. All the subjects were randomly assigned to three groups, Low-Rem group (low dose remimazolam, 0.15 mg/kg, iv, *n* = 75), High-Rem group (high dose remimazolam, 0.2 mg/kg, iv, *n* = 75), and Propofol group (propofol 2 mg/kg, iv, *n* = 75). Every individual in this trial was given nalbuphine hydrochloride (0.2 mg/kg, iv) before administration of remimazolam or propofol. The primary outcome was the success rate of sedation. Haemodynamic parameters and adverse events were recorded to evaluate safety. Satisfaction of sedation from patients, anesthesiologists and gastroenterologists were also recorded.

**Results:**

The success rate of colonoscopy procedure was 100% in both High-Rem and Propofol groups, but it was 89% in Low-Rem group (*p* < 0.05). Furthermore, the induction time of anesthesia was shorter in Propofol group, when compared to the Low-Rem group and the High-Rem group (*p* < 0.05). The recovery time in Low-Rem group, High-Rem group, and Propofol group was 2.33, 2.43, and 3.21 min (*p* < 0.05) respectively, and the time of discharge was 25.00, 25.01, and 27.56 min (*p* < 0.05) respectively. Simultaneously, the incidence of adverse events such as hypotension, bradycardia, and respiratory depression in the remimazolam groups were significantly lower than that in the propofol group. No significant differences were observed among the three groups in Ramsay scale, BPS-NI scale, and Limb movement classification. Moreover, patients, anesthesiologists, and gastroenterologists were all satisfied with the sedation process.

**Conclusion:**

Remimazolam can be used safely and effectively for colonoscopy. 0.2 mg/kg remimazolam and propofol have the same sedation success rate and more stable hemodynamics and fewer side effects than propofol.

**Clinical trial registration:**

ChiCTR2100054053.

## 1 Introduction

Colonoscopy is a common technique for the diagnosis and treatment of lower gastrointestinal diseases in gastroenterology department. However, since colonoscopy is an invasive procedure, patients, when without adequate sedation, usually tolerate the procedure poorly because of pain, discomfort and scare (1). Therefore, colonoscopy is generally performed under moderate to deep sedation with adequate analgesia to ensure the success of the procedure and effective control of patients’ pain and discomfort, allow doctors to use the technique more widely. Currently, midazolam and propofol are the main sedation drugs in clinical colonoscopy. Midazolam, a drug belonging to benzodiazepine group, has a strong amnestic and sedative effect. However, it has a long duration of action and slow recovery from anesthesia (2). Propofol is widely used for the induction and maintenance of general anesthesia and sedation due to its fast onset, short duration of action, and easy titration, but hypotension, respiratory depression and pain at the injection site are complications that limit its utilization (3).

Remimazolam is a new type of ultra-short-effect benzodiazepine that acts via gamma-aminobutyric acid (GABA) receptors (4). Since its application in clinic, rapid onset and sedative offset of remimazolam have been highlighted in several clinical trials as a programmed sedative (5–7). The adverse event spectrum of remimazolam was superior to that of propofol group in terms of hemodynamic fluctuation, excessive sedation depth, and low SpO_2_, and the much lower incidence of injection pain (8). However, there was few clinical trials on the application of remimazolam in painless colonoscopy. The purpose of this study was to investigate the sedation efficacy and safety of two different doses of remimazolam in adult colonoscopy.

## 2 Materials and methods

This was a prospective, randomized, double-blind, and different-dose remimazolam study that included adults scheduled for colonoscopy procedures. The study adheres to the 2010 Consolidated Standards of Reporting Trials (CONSORT) statement (9). The trial got its ethical approval from the Ethics Committee of Ji’an Hospital, Shanghai East Hospital [No. 2020 (08)], and has been registered with the Chinese Clinical Trial Registry (ChiCTR2100054053). All patients signed an informed consent form before enrollment in the study.

### 2.1 Participants

During the study period from Sep. 2021 to Mar. 2022, 248 patients were screened and 225 participants were enrolled. Inclusion criteria included: (1) patients undergoing painless colonoscopy; (2) aged 18–80 years; (3) body mass index (BMI)18.5 ~ 30 kg/m^2^; (4) those with American Society of Anesthesiologists (ASA) Physical Status I to III. Exclusion criteria included: (1) those who refused to participate in the trial; (2) those diagnosed with severe heart, brain, lung, liver, kidney or metabolic diseases; (3) those whose time of colonoscopy did not exceed 15 min; (4) ECG prompt: heart rate < 50 beats/min; (5) a history of acute inflammation of the respiratory tract that had not been cured within 2 weeks; (6) preoperative hypertension with systolic blood pressure (SBP) ≥180 mmHg and (or) diastolic blood pressure (DBP) ≥110 mmHg, or hypotension <90/60 mmHg; (7) neuromuscular system diseases and mental diseases; (8) those who had anemia or thrombocytopenia; (9) suspected abuse of narcotic analgesics or sedatives; (10) those who were predicted to have or have had difficult airway; (11) those who did not cooperate and could not communicate; (12) those with allergies or contraindication to benzodiazepines, opioids, propofol, and lidocaine; (13) the painless colonoscopy procedure lasted more than 15 min.

### 2.2 Study design

The patients were randomly in a 1:1:1 ratio divided into Group Low-Rem (remimazolam 0.15 mg/kg), Group High-Rem (remimazolam 0.2 mg/kg) and Group Propofol (propofol 2 mg/kg) by the random number table method. The randomization sequence was placed in sequentially numbered opaque sealed envelopes and revealed by another investigator after the participants’ enrollment. Since nature of the propofol emulsion is easily recognizable, it was wrapped in white adhesive tape. All patients and the endoscopy suite nurse (the administrator) were unaware of the medication given. The anesthesiologists and the gastroenterologists were unaware of the medication and dosage given during the entire procedure. The anesthesiologists monitored the patients and recorded the data.

In this trial, all patients fasted for at least 8 h and 2 h of water before painless colonoscopy. They were given intravenously nalbuphine hydrochloride injection (Yichang Human Well Pharmaceutical Co. Ltd., Hubei, China), a bolus dose of 0.2 mg/kg, 1 minute before the procedure. They were given an initial intravenous dose of remimazolam (Yichang Human Well Pharmaceutical Co. Ltd., Hubei, China) 0.15 mg/kg (Low-Rem group), remimazolam 0.2 mg/kg (High-Rem group), or propofol (AstraZeneca Pharmaceuticals Co. Ltd., China) 2 mg/kg (Propofol group). The colonoscopy procedure was started when the required sedation level was reached (Modified Observer’s Assessment of Alertness/Sedation, MOAA/S ≤ 2) and the score scale was described as 6: Agitated; 5: Ready response to name spoken in normal tone(alert); 4: Lethargic response to name spoken in normal tone; 3: Respond only after the name is called loudly and/or repeatedly; 2: Respond only after mild prodding or shaking; 1: No response to mild prodding or shaking; 0: No response to deep stimulus (10).

If sedation was deemed to be inadequate (MOAA/S > 2) or the colonoscopy procedure failed, up to a maximum of five supplemental doses administered as IV boluses remimazolam 0.04 mg/kg or propofol 0.4 mg/kg, were permitted 1 min after the end of the initial dose, with a one-minute interval in between. If the initial dose and the supplemental doses were not sufficient to obtain adequate sedation for scope insertion, additional propofol of 0.4 mg/kg per time, as a sedative rescue medication, was administered at the start of the procedure at the investigator’s discretion. For all patients, supplemented oxygen (2 L/min) was administered through a nasal cannula. If there was a decrease in oxygen saturation, the oxygen flow rate was increased by the anesthesiologist and the mandible was lifted bilaterally. Mask ventilation and tracheal intubation were used to assist with breathing if necessary. Hypotension was defined when systolic blood pressure (SBP) was <90 mmHg or when a 20% decrease in basal value occurred and lasted for ≥1 min, then ephedrine (6 mg, IV) was administered. Heart rate (HR) <50 bpm was defined as bradycardia and if that occurred, atropine (0.5 mg, IV) was used. On completion of this process, patients were sent to the post-anesthesia care unit (PACU) for further observation.

### 2.3 Measurements

The primary outcome was the success rate of sedation, which was defined as: (1) completion of the whole endoscopy procedure; (2) no requirement for an alternative and/or rescue sedative; (3) administration of up to a maximum of 5 supplemental doses within 15 min after the initial dose.

The secondary outcomes were as follows:

1. Haemodynamic parameters included SBP, diastolic blood pressure (DBP), mean arterial pressure (MAP), HR, and Pulse Oximeter Oxygen Saturation (SpO_2_) were recorded in all groups after admission (T0), 1 min after first administration (T1), 3 min after first administration (T2), 5 min after first administration (T3), 10 min after first administration (T4), 15 min after first administration (T5), 20 min after first administration (T6) during colonoscopy.
2. Adverse events included: (1) incidence of bradycardia; (2) incidence of hypoxia (oxygen saturation < 90%, which lasted continuously for ≥1 min or any necessary medical interventions); (3) incidence of nausea and/or vomiting, vertigo and belching after the operation.
3. Anesthesia induction time: defined as the time interval from the initial administration of the trial drug to the first MOAA/S score of 3 or less.
4. Duration time: defined as the time from the entry of the endoscope into the external anal to the end of the examination when the endoscope exited the external anal.
5. Recovery time: defined as the time from the discontinuation of the sedative medication to full alertness (the first of three consecutive MOAA/S score of 5).
6. Discharge time: defined as the time from the end of administration of drugs to meet the discharge criteria (modified postanesthesia discharge scoring system score of at least 9, with two points in the vital sign item (11)).
7. The initial doses of remimazolam or propofol and the number of top-up doses needed to maintain adequate sedation.
8. Comparison of other sedation evaluating indicators, such as Ramsay scale (12), Behavioural Pain Scale Non-Intubated (BPS-NI) score (13), and limb movement classification (Grade1, finger and toe joint movement; Grade 2, wrist and ankle joint movement; Grade 3, knee and elbow joint movement that affected the operation and needed additional sedative drugs; Grade 4, myelinated joint movement that affected the operation and needed additional sedative drugs).
9. Satisfaction measurement of patients, anesthesiologists, and gastroenterologists using the following scale: 1 to 3 indicating “dissatisfied,” 4 to 6 indicating “satisfied,” and 7 to 10 indicating “very satisfied”.

### 2.4 Sample size

The sample size was calculated based on the incidence of hypotension in all three groups with Pass software version 15.0 (NCSS, Kaysville, UT, United States). According to Borkett’s trial, the sedation success rate in the Low-Rem group was 56%, and the High-Rem group was 64%. We assumed that the sedation success rate in the Propofol group was 99%, considering the first type of error, *α* value of 0.05, power of 90%, 72 patients were required for each group. Considering the dropout rate of 10%, 240 patients were enrolled in three groups.

### 2.5 Statistics

All data were assessed for normal distribution using the Shapiro–Wilk test. The data were expressed as mean ± standard deviations (SD) for normally distributed data or median with interquartile range for data not normally distributed. Data homogeneity of variance was evaluated by using the Levene test. Intergroup comparisons of continuous variables were performed using ANOVA or Kruskal-Wallis test. Between-group comparisons of categorical variables were analyzed by chi-square of Fisher’s exact tests. *p* < 0.05 was considered to be statistically significant. All data were performed using GraphPad Prism (v 9.1.1, *GraphPad Prism*).

## 3 Results

**Figure 1:**
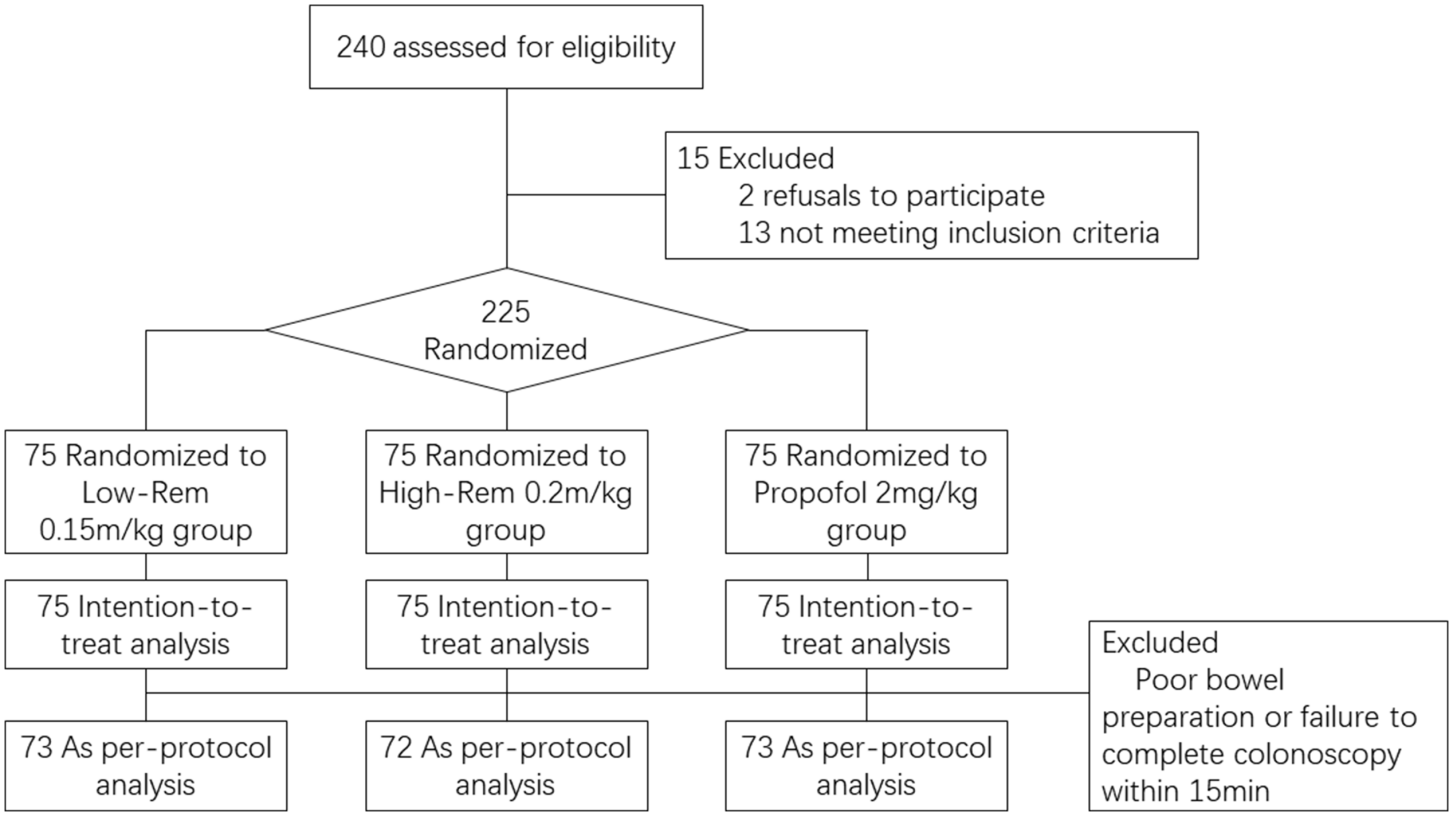
Specific flow diagram of patient selection.

During the study period from Sep. 2021 to Mar. 2022, a total of 240 patients who underwent painless colonoscopy were evaluated for eligibility before enrollment. 15 of them were excluded (3 were excluded due to frequent premature ventricular contractions, 4 were excluded due to respiratory infection within 2 weeks, 3 were excluded due to severe hypertension, 3 were excluded due to long term use of sleeping pills, and 2 was excluded due to refusal to participate in the study). 225 patients were recruited for our study and were prospectively randomized at the ration of 1:1:1 into the groups of Low-Rem (*n* = 75), High-Rem (*n* = 75) and Propofol (*n* = 75). After the patients were randomized, 2 cases in Low-Rem group were excluded due to poor bowel preparation or failure to complete colonoscopy within 15 min. 3 cases in High-Rem group and 2 cases in Propofol group were excluded for the same reason, respectively. Finally, 73 patients were in Low-Rem group, 72 patients were in High-Rem group and 73 patients were in Propofol group. A specific flow diagram of the patient selection is presented in Figure 1.

### 3.1 Demographic characteristics

There lists the demographics and baseline characteristics of the patients from each group. There was no statistically significant difference in sex, age, weight, height, BMI, ASA classification, and. The three groups were well matched at baseline (Table 1).

**Table 1:** The demographic characteristics of the patients.

Group	Low-Rem (*N* = 73)	High-Rem (*N* = 72)	Propofol (*N* = 73)	*F* value/*χ*^2^ value	*p*- value
Age, year	57.97 ± 10.50	56.60 ± 9.94	60.27 ± 10.70	2.323	0.100
Gender				3.31	0.191
Male, n (%)	37 (50.7)	39 (54.2)	29 (39.7)		
Female, n (%)	36 (49.3)	33 (45.8)	44 (60.3)		
Height, cm	164.58 ± 8.37	162.97 ± 7.77	163.85 ± 7.78	0.734	0.481
Weight, kg	63.43 ± 11.58	62.13 ± 9.15	61.88 ± 9.76	0.486	0.616
BMI, kg/m^2^	23.28 ± 2.88	23.32 ± 2.52	22.93 ± 2.38	0.491	0.613
ASA classification, n (%)				0.316	0.854
I	49 (67.1)	49 (68.1)	52 (71.2)		
II	24 (32.9)	23 (31.9)	21 (28.8)		
III	0	0	0		

The values are presented as numbers (%). ASA, American Society of Anesthesiologists; DBP, diastolic blood pressure; HR, heart rate; SBP, systolic blood pressure; SpO_2_, oxygen saturation measured by pulse oximetry.

### 3.2 Primary outcome

The sedation success rate was 65 of 73 (89%) in the Low-Rem group, 72 of 72 (100%) in the High-Rem group, and 73 of 73 (100%) in the Propofol group. The sedation success rate in the Low-Rem group was significantly lower in comparison to the other two groups (*p* < 0.001; Table 2). Patients who have failed sedation in the Low-Rem group received sedative rescue medication or multiple additional drugs to complete the colonoscopy.

**Table 2:** Primary outcomes.

Group	Low-Rem (*N* = 73)	High-Rem (*N* = 72)	Propofol (*N* = 73)	*χ*^2^ value	*p*- value
Sedation success rate, n (%)	65 (89)	72 (100.0) ^#^	73 (100.0) ^#^	16.496	0.000
Completion of Colonoscopy, n (%)	73 (100.0)	72 (100.0)	73 (100.0)	/	/
More than five total doses of study medication within any 15-min interval, n (%)	5 (6.8)	0 (0)	0 (0)	10.165	0.006
Administration of rescue medication (%)	22 (30.1)	0 (0) ^#^	0 (0) ^#^	48.604	0.000

The values are presented as numbers (%). ^#^*p* < 0.05 compared with Low-Rem group. ^*^p < 0.05 compared with High-Rem group.

### 3.3 Secondary outcomes

**Figure 2:**
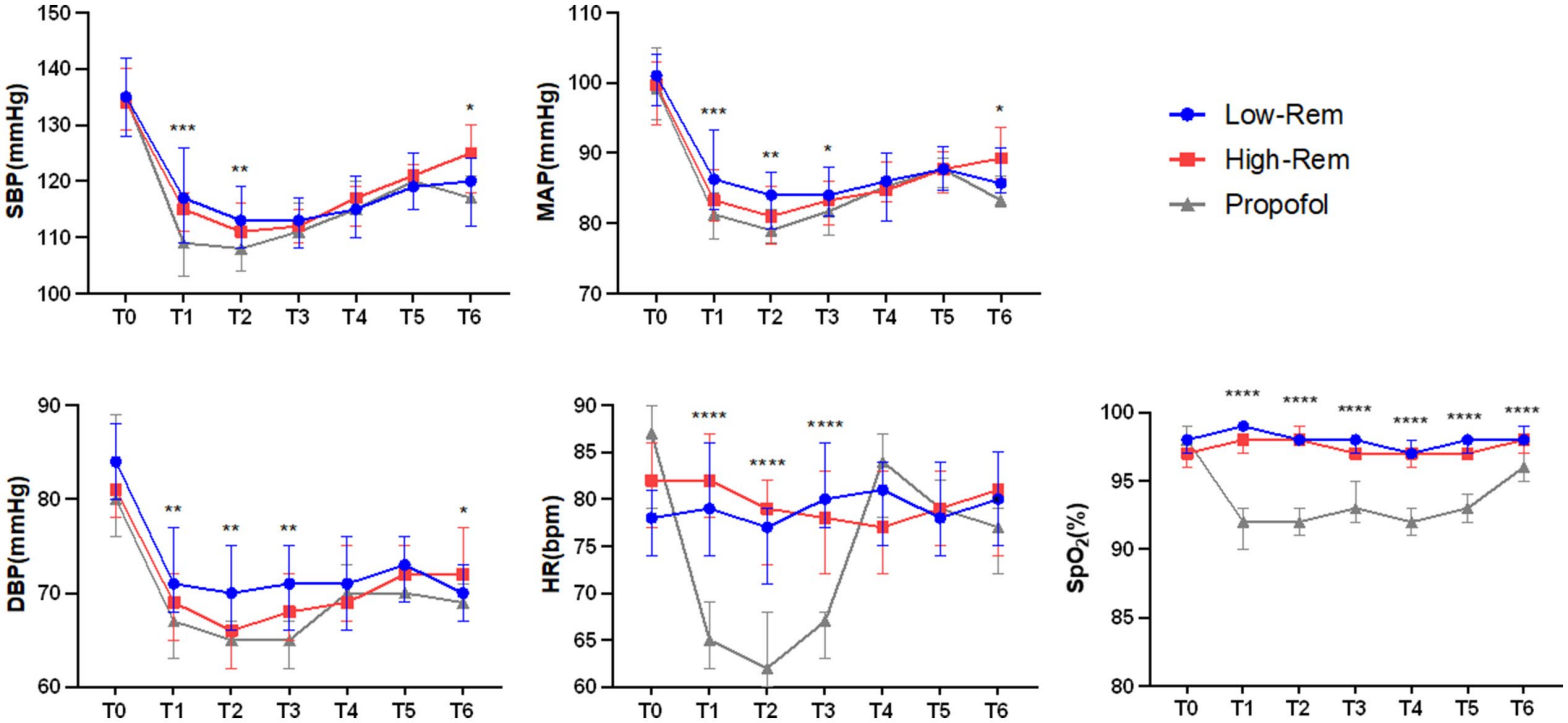
Haemodynamic parameters fluctuation during colonoscopy at time after admission (T0), 1 min after first administration (T1), 3 min after first administration (T2), 5 min after first administration (T3), 10 min after first administration (T4), 15 min after first administration (T5), 20 min after first administration (T6) during colonoscopy. Systolic blood pressure (SBP), diastolic blood pressure (DBP), mean arterial pressure (MAP), heart rate (HR), and Pulse Oximeter Oxygen Saturation (SpO_2_). The values are presented as either mean ± SD, ^*^p < 0.05, ^**^p < 0.01, ^***^p < 0.001 compared with Propofol group.

The incidence of hypotension was significantly lower in Low-Rem group (7%) and in High-Rem group (10.7%), when compared with Propofol group (26.8%) (*p* < 0.05; Table 3). The specific haemodynamic comparison results were detailed in Figure 2. The decrease in SBP was significantly higher in the propofol group than in the Low-Rem group at T1 and T2, and the decrease in SBP was higher in the propofol group than in the High-Rem group at T6 (*p* < 0.05). Moreover, the decrease in DBP was significantly higher in the propofol group than in the Low-Rem group at T1, T2 and T3, and the decrease in DBP was higher in the propofol group than in the High-Rem group at T6 (*p* < 0.05). Also, the decrease in MAP was significantly higher in the propofol group than in the Low-Rem group at T1, T2 and T3, and the decrease in MAP was higher in the propofol group than in the High-Rem group at T6 (*p* < 0.05). HR was significantly decelerated in the propofol group compared to the other two groups at T1, T2 and T3 (*p* < 0.05). SpO_2_ was significantly reduced in the propofol group compared to the other two groups from T1 to T6 (Figure 2).

**Table 3:** Adverse events.

Group	Low-Rem (*N* = 73)	High-Rem (*N* = 72)	Propofol (*N* = 73)	*p*- value	*χ*^2^ value
Hypotension, *n* (%)	5 (7.0)	8 (10.7)	20 (26.8)^*#^	0.001	13.353
Bradycardia, *n* (%)	1 (1.4)	4 (5.6)	13 (17.8) ^#^	0.001	14.056
Respiratory depression / Apnea, n (%)	1 (1.4)	3 (4.2)	11 (15.1) ^#^	0.003	11.926
Nausea and vomiting, *n* (%)	0	0	2 (2.7)	0.135	4.009
Vertigo, *n* (%)	0	1 (1.4)	4 (5.5)	0.071	5.283
Belching, *n* (%)	0	0	3 (4.1)	0.049	6.042

The values are presented as numbers (%). ^#^*p* < 0.05 compared with Low-Rem group. ^*^p < 0.05 compared with High-Rem group.

The incidence of bradycardia (17.8%) and respiratory depression (15.1%) were significantly higher in Propofol group than in Low-Rem group and in High-Rem group (*p* < 0.05; Table 3). No significant differences were observed in incidence of other adverse events, such as nausea, vomiting, vertigo, and belching (Table 3).

In the Propofol group, the induction time was shorter, however, the recovery time and discharge time were longer than in the Low-Rem and High-Rem groups (*p* < 0.05). No significant differences were observed in the duration time among the three groups (*p* > 0.05; Table 4).

**Table 4:** Comparison of induction time, duration time, recovery time, discharge time and dosage of medication.

Group	Low-Rem (*N* = 73)	High-Rem (*N* = 72)	Propofol (*N* = 73)	*F* value	*p* value
Induction time, min	1.84 ± 0.68	1.77 ± 0.83	1.47 ± 0.71^#**^	5.170	0.006
Duration time, min	10.00 ± 3.54	10.08 ± 3.92	10.68 ± 6.27	0.453	0.636
Recovery time, min	2.33 ± 1.80	2.43 ± 1.61	3.21 ± 2.18^##*^	4.743	0.01
Discharge time, min	25.00 ± 8.83	25.01 ± 5.71	27.56 ± 8.10^#*^	2.693	0.07
Initial dose, mg	9.05 ± 1.90	10.62 ± 2.11	121.88 ± 28.73	1092.022	0
Supplemental times	2.68 ± 1.69	1.36 ± 1.17^###^	1.15 ± 0.97^###^	29.328	0.000
Supplemental dose, mg	6.61 ± 4.75	3.15 ± 2.73	26.76 ± 21.21	73.547	0.000

The values are presented as either mean ± SD or numbers (%). ^#^*p* < 0.05; ^##^*p* < 0.01; ^###^*p* < 0.001 compared with Low-Rem group. ^*^p < 0.05; ^**^p < 0.01; ^***^p < 0.001 compared with High-Rem group.

The sedation depth during colonoscopy was assessed using Ramsay Scale, BPS-NI scale, and Limb movement classification. No significant differences were found among the three groups in Ramsay Scale and BPS-NI scale. The probability of limb movement was higher in the Low-Rem group than in the High-Rem and Propofol groups. In all the three groups, patient satisfaction was 100% and indicated willingness to undergo the same sedation protocol regimen in the future. Gastroenterologists and anesthesiologist in all treatment groups were slightly less satisfied than in the other two groups (Table 5).

**Table 5:** Anesthesiologist’s, gastroenterologist’s and patient’s evaluation about the performance of the sedation.

Group	Low-Rem (*N* = 73)	High-Rem (*N* = 72)	Propofol (*N* = 73)	*p* value	*χ*^2^ value
Ramsay scale, *n* (%)				0.160	3.662
1–2 score	0	0	0		
3–4 score	7 (9.6)	3 (4.2)	2 (2.7)		
5–6 score	66 (90.4)	69 (95.8)	71 (97.3)		
BPS-NI score, *n* (%)				0.128	7.158
1–4 score	63 (86.3)	68 (94.4)	70 (95.9)		
5–8 score	8 (11.0)	4 (5.6)	3 (4.1)		
9–12 score	2 (2.7)	0	0		
Limb movement classification, *n* (%)				0.037	10.195
1	63 (86.3)	69 (95.8)	70 (95.9)		
2	3 (4.1)	1 (1.4)	3 (4.1)		
3	7 (9.6)*	2 (2.8)	0		
4	0	0	0		
Patients, *n* (%)					
Satisfied (7–10)	73 (100.0)	72 (100.0)	73 (100.0)		
Gastroenterologists, *n* (%)					
Satisfied (7–10)	68 (93.2)*	72 (100.0)	73 (100.0)	0.017	8.207
Anesthesiologist, *n* (%)					
Satisfied (7–10)	70 (95.9)*	72 (100.0)	73 (100.0)	0.049	6.042

The values are presented as numbers (%). *p < 0.05, compared with Propofol group.

## 4 Discussion

This study indicated that remimazolam 0.2 mg/kg showed the advantages of rapid onset, more stable hemodynamics, better sedation quality and depth, and fewer adverse reactions in adult colonoscopy patients.

The sedation rate of remimazolam single drug was insufficient. As reported by Borkett et al. (5), the success rates of sedation with remimazolam alone at doses of 0.10, 0.15, and 0.20 mg/kg in adult patients were 32, 56, and 64%, respectively. In other words, the dose required to achieve sedation success with remimazolam alone is larger. The success rate of sedation in 0.2 mg/kg remimazolam group was 82%, in 0.3 mg/kg group was 98%, and in 0.4 mg/kg group was 96% (14). However, when combined with small doses of opioids, the sedation rate of remimazolam was significantly improved. The sedation success rate of the 0.15 mg/kg remimazolam group was 88.5%, and 0.2 mg/kg remimazolam was 98.7% in Zhu’s trial when combined with 5 μg sufentanil (15). It was 100% in 0.10 or 0.20 mg/kg remimazolam tosilate group when combined with 0.01 mg/kg butorphanol in Tan’s report (16). In our study, 0.2 mg/kg nalbuphine was used in combination, and the success rate of sedation was 89% in 0.15 mg/kg remimazolam group, 100% in 0.2 mg/kg remimazolam group and 2 mg/kg propofol group. Opioids synergize the sedation of remimazolam, so compounding different opioids and different dosages will affect the sedation success of remimazolam to varying degrees.

Propofol were used for gastrointestinal endoscopy sedation for about 20 years, its advantages over benzodiazepines include rapid onset of action, rapid recovery, without residual sedative effects or anterograde amnesia (17). However, it has significant inhibitory effects on hemodynamics and respiration in a dose-dependent manner (18–20). In our study, the incidence of hypotension was 26.8%, bradycardia was 17.8% and respiratory depression was 15.1%. Food and Drug Administration (FDA) has already granted approval for remimazolam use in procedural sedation (21). It has already been shown that remimazolam overcame inhibitory effects on hemodynamics and respiration with a favorable safety profile (22). Similarly, Barbosa (23) conducted a systematic review and meta-analysis which concluded remimazolam had clinically similar efficacy and greater safety when compared with propofol for sedation in gastrointestinal endoscopies. Remimazolam was associated with significantly lower rates of respiratory depression, hypotension, hypotension requiring treatment, and bradycardia. Zhang (7) compared remimazolam with propofol for hysteroscopy and found that the incidence of hypotension, low SpO_2_ and bradycardia in propofol group was much higher than that in remimazolam group. In our study, significant haemodynamic fluctuations in the three groups occurred mainly at 1, 3 and 5 min after induction, with the lowest fluctuations in the Low-Rem group and the highest in the Propofol group. The alteration of HR by propofol was obvious and significant, and was not significantly different between 0.15 mg/kg remimazolam and 0.2 mg/kg remimazolam. The incidence of respiratory depression in patients who received remimazolam was lower than that in the propofol group.

Sedation effectiveness was dose-dependent of remimazolam (24), however, as the dose is progressively increased, the incidence of adverse reactions by remimazolam are also significantly increased, such as vertigo and prolonged sedation recovery time (14). Here, in our study, the induction time, the recovery time, and the discharge time had no difference between Low-Rem group and High-Rem group. Yet, the induction time was prolonged, the recovery time and the discharge time were shortened compared to the propofol group. The induction time was 1.84 and 1.77 min in the 0.15 and 0.20 mg/kg remimazolam groups, respectively, compared with the propofol group (1.47 min) (*p* < 0.01). The recovery time was 2.33 to 2.43 min for different doses of remimazolam groups as compared with 3.21 min for propofol group (*p* < 0 0.05). The significant decrease in recovery time appears to result from high clearance, small volume of distribution, and susceptibility to ester hydrolysis by carboxylesterase-1 to an inactive carboxylic acid metabolite of remimazolam (22). The duration time had no difference among three groups.

In addition, the criteria used to assess the depth of sedation were various from study to study. MOAA/S as a commonly used score to assess the level of sedation in patients, was universally used in gastroenteroscopy. Painlessness is defined as no pain felt by the patient. The intensity of colonoscopic stimulation is not very high. When the MOAA/S ≤ 2 points, the patient does not feel pain after awakening, so we defined this criterion, consistent with Cui’s (14), Zhang’s (25), and Zhu’s (15). Different studies used different sedation scores, such as MOAA/S ≤ 3 points (26–28), MOAA/S ≤ 1 points (16, 29, 30), and the sedation results naturally fluctuated. Moreover, we also compared three other methods of sedation assessment such as the Ramsay scale, BPS-NI score, and limb movement to evaluate intraoperative sedation and analgesia. Ramsay scale (31) and BPS-NI score (32) have been used for patients with dementia or in critically ill intensive care unit (ICU) patients, it may possibly be used for patients unable to communicate or patients with unconcious. Limb movement classification is helpful in evaluating the degree of sedation and analgesia and estimating additional sedative medications need to be administrated (33). When using the Ramsay scale and BPS-NI for sedation evaluation in the three groups, although there was no statistical difference, the Low-Rem group was lower than the High-Rem group and the Propofol group, and the High-Rem group and the Propofol group were similar. When using the Limb movement for sedation evaluation, the Low-Rem group was significantly lower than the Propofol. When Limb movement was used for sedation evaluation, it was significantly lower in the Low-Rem group than in the Propofol group, while there was no significant difference between the High-Rem and Propofol groups. It can be seen that the MOAA/S index is more sensitive when used for sedation evaluation in the three groups. The 0.15 mg/kg remimazolam group resulted in a decrease in satisfaction of gastroenterologists and anesthesiologists compared to the other two groups due to inadequate sedation and limb movement. Remimazolam of 0.2 mg/kg contributes to the rapid awakening and conscious discharge of outpatients.

In conclusion, the present study shows 0.2 mg/kg remimazolam is non-inferior to propofol in terms of sedation during colonoscopy, and has more stable hemodynamics and fewer side effects than propofol.

### 4.1 Limitations

There are some limitations in this study. We studied only the efficacy and safety of remimazolam in painless colonoscopy. Gastroenteroscopy will undoubtedly require more time and greater medications than colonoscopy. The efficiency of sedation after prolonged or sustained use of remimazolam needs to be further investigated. On the other hand, gastrointestinal endoscopy usually requires periodic re-examination, and we still do not know whether cognitive dysfunction or memory deficiency may occur after repeated multiple uses of remimazolam. In addition, this is a single-centre study, with a mean patient age of only 60 years and remimazolam doses of just 0.15 and 0.2 mg/kg, further studies on the efficacy and safety of remimazolam including dose optimisation, age stratification, larger sample sizes and multicentre collaborations are needed.

## 5 Conclusion

Remimazolam is a safe and effective sedative for patients undergoing colonoscopy. 0.2 mg/kg remimazolam is equivalent to propofol for sedation when combined with small doses of opioids.

## Data Availability

The original contributions presented in the study are included in the article/supplementary material, further inquiries can be directed to the corresponding author/s.
